# GWAS of lipids in Greenlanders finds association signals shared with Europeans and reveals an independent *PCSK9* association signal

**DOI:** 10.1038/s41431-023-01485-8

**Published:** 2023-10-30

**Authors:** Ninna Karsbæk Senftleber, Mette K. Andersen, Emil Jørsboe, Frederik Filip Stæger, Anne Krogh Nøhr, Genis Garcia-Erill, Jonas Meisner, Cindy G. Santander, Renzo F. Balboa, Arthur Gilly, Peter Bjerregaard, Christina Viskum Lytken Larsen, Niels Grarup, Marit Eika Jørgensen, Eleftheria Zeggini, Ida Moltke, Torben Hansen, Anders Albrechtsen

**Affiliations:** 1 Clinical Research, Copenhagen University Hospital—Steno Diabetes Center Copenhagen, Herlev, Denmark; 2 Section for Computational and RNA Biology, Department of Biology, University of Copenhagen, Copenhagen, Denmark; 3 Novo Nordisk Foundation Center for Basic Metabolic Research, Faculty of Health and Medical Sciences, University of Copenhagen, Copenhagen, Denmark; 4 Big Data Institute, Li Ka Shing Centre for Health Information and Discovery, University of Oxford, Oxford, United Kingdom; 5 Nuffield Department of Population Health, University of Oxford, Oxford, UK; 6 Center for Clinical Data Science, Department of Clinical Medicine, Aalborg University and Research, Education, and Innovation, Aalborg University Hospital, Aalborg, Denmark; 7 Novo Nordisk Foundation Center for Protein Research, Faculty of Health and Medical Sciences, University of Copenhagen, Copenhagen, Denmark; 8 Institute of Translational Genomics, Helmholtz Zentrum München – German Research Center for Environmental Health, Neuherberg, Germany; 9 Centre for Public Health in Greenland, National Institute of Public Health, University of Southern Denmark, Copenhagen, Denmark; 10 Greenland Center for Health Research, Institute for Health and Nature, University of Greenland, Nuuk, Greenland; 11 Steno Diabetes Center Greenland, Nuuk, Greenland; 12 Technical University of Munich (TUM) and Klinikum Rechts der Isar, TUM School of Medicine, Munich, Germany

**Keywords:** Genome-wide association studies, Risk factors, Dyslipidaemias

## Abstract

Perturbation of lipid homoeostasis is a major risk factor for cardiovascular disease (CVD), the leading cause of death worldwide. We aimed to identify genetic variants affecting lipid levels, and thereby risk of CVD, in Greenlanders. Genome-wide association studies (GWAS) of six blood lipids, triglycerides, LDL-cholesterol, HDL-cholesterol, total cholesterol, as well as apolipoproteins A1 and B, were performed in up to 4473 Greenlanders. For genome-wide significant variants, we also tested for associations with additional traits, including CVD events. We identified 11 genome-wide significant loci associated with lipid traits. Most of these loci were already known in Europeans, however, we found a potential causal variant near *PCSK9* (rs12117661), which was independent of the known *PCSK9* loss-of-function variant (rs11491147). rs12117661 was associated with lower LDL-cholesterol (β_SD_(SE) = −0.22 (0.03), *p* = 6.5 × 10^−12^) and total cholesterol (−0.17 (0.03), *p* = 1.1 × 10^−8^) in the Greenlandic study population. Similar associations were observed in Europeans from the UK Biobank, where the variant was also associated with a lower risk of CVD outcomes. Moreover, rs12117661 was a top eQTL for *PCSK9* across tissues in European data from the GTEx portal, and was located in a predicted regulatory element, supporting a possible causal impact on *PCSK9* expression. Combined, the 11 GWAS signals explained up to 16.3% of the variance of the lipid traits. This suggests that the genetic architecture of lipid levels in Greenlanders is different from Europeans, with fewer variants explaining the variance.

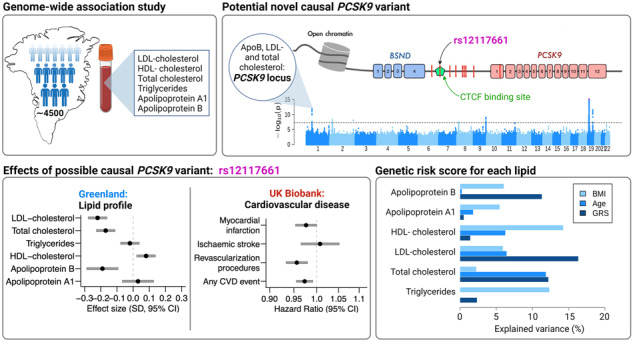

## Introduction

Cardiovascular disease (CVD) is a major health burden worldwide, and ischaemic heart disease and stroke have been estimated to account for more than one-fourth of all deaths in 2016, making these diseases the leading cause of death globally [1]. Currently, one of the most successful strategies for preventing CVD is to treat dyslipidemia with lipid-lowering drugs [2]. Circulating levels of blood lipids are affected by lifestyle, diet, and genetic variation [3]. The genetics of circulating lipid levels has been studied widely, resulting in the identification of more than 940 loci in genome-wide association studies (GWAS) of levels of triglycerides, LDL-, HDL-, non-HDL-, and total cholesterol in more than 1.65 million individuals from different ancestry groups [4, 5]. In Europeans, each of the identified variants only explains a small fraction of the trait variance, and when combining up to 2 million variants in a genetic risk score (GRS) up to 22% of the variance could be explained [4, 6, 7].

A key finding from a European GWAS of lipid levels is the discovery of a causal *PCSK9* loss-of-function (LoF) variant (p.Arg46Leu; rs11591147) [8], which has led to the development of *PCSK9*-inhibitors to treat dyslipidemia [9]. Besides this *PCSK9* LoF variant (rs11591147), an independent signal in the *PCSK9* region, driven by the rare *PCSK9* p.Asn157Lys missense variant (rs143117125), has been reported in a cohort of more than 69,000 Norwegians [10].

Although the most recent genetic studies of circulating lipid levels include individuals of different ancestries, the majority are still of European ancestry [4, 5]. Hence, additional genetic variants contributing to the variation in lipid levels could be identified by studying other ancestry groups. In this respect, the Greenlandic population could be of particular interest, because previous studies have shown that the genetic architecture of metabolic diseases in this population differs markedly from that in European populations [11, 12] and because an Arctic specific variant with a very high impact on LDL-cholesterol levels and CVD risk has been identified [13, 14]. Moreover, the prevalence of high LDL-cholesterol, as well as cardiometabolic diseases, in Greenland is high [15–18]. In a recent population survey, more than 70% of the 45–54-year-old participants had LDL-cholesterol levels above 3 mmol/L, which is considered too high for individuals with no other CVD risk factors [18]. Motivated by these observations, we aimed to investigate the genetics of triglycerides, LDL-cholesterol, HDL-cholesterol, total cholesterol, apolipoprotein A1 (apoA1), and apolipoprotein B (apoB) in Greenlanders. Furthermore, we aimed to describe the genetic architecture of these six lipid traits in Greenlanders compared to Europeans, and to assess if the lipid-associated genetic variants affected the risk of CVD.

## Materials and methods

### Study population

We used data from three Greenlandic cohorts; The *Inuit Health in Transition* (IHIT, 2005–2010), comprising 3115 individuals living in Greenland [19], the *Greenland population study* (B99) and BBH cohorts (1998–2001) comprising 1401 individuals living in Greenland, and 503 Greenlanders living in Denmark, respectively [20]. There was an overlap of individuals between IHIT and B99 (*N* = 295), and these were assigned to B99. By combining the three cohorts, we were able to include up to 4473 individuals in the analyses.

### Biochemical measurements

Blood samples were collected from fasting participants in IHIT [19] as well as the majority of participants of B99, and in fasting and non-fasting participants of BBH. Serum concentrations of total cholesterol, HDL-cholesterol, triglycerides, apoA1, and apoB were measured and concentrations of LDL-cholesterol were calculated. Triglyceride measurement and the calculation of LDL-cholesterol was only done for fasting participants [21]. Participants also had anthropometric measures and blood pressure taken, as well as an oral glucose tolerance test for those aged ≥35 years in B99 and BBH and for those aged ≥18 years in IHIT, from whom blood samples were also taken 2 h after the glucose ingestion to measure plasma glucose, serum insulin, and serum C-peptide, as previously described [19, 20].

### Cardiovascular disease outcomes

We obtained information on ischaemic stroke, myocardial infarction, and revascularization procedures from The Greenland National Patient register, the Danish National Patient Register, and the causes of death register from both Greenland and Denmark. We used the International Statistical Classification of Diseases and Related Health Problems- (ICD-) 8, ICD10, and International Classification of Primary Care 2 (ICPC-2) codes, as well as The Classification of Operations and Treatments and The Nordic Medico-Statistical Committee (NOMESCO) Classification of Surgical Procedures for procedures (Supplementary Tables 1, 2). Follow-up data in registries were only available for IHIT and B99, resulting in the inclusion of 4110 individuals in the analyses of CVD events.

### Genotyping

All samples were genotyped on the Multi-Ethnic Global Array (MEGA chip, Illumina), with ~2 M variants. This chip was chosen, to maximise the capture of genetic variation in Inuit since this chip also includes variants selected in East Asian and Indigenous Americans. Genotypes were called jointly for all cohorts using the GenCall module of the GenomeStudio software (Illumina). The data set underwent quality control, removing duplicate samples, individuals missing >5% genotypes, and variants with >1% missing genotypes. In total, data from 4607 individuals and 1,769,375 variants passed the quality control.

### Imputation

To create a reference panel, we obtained short paired-end whole genome sequencing data (Illumina) for 447 Greenlanders. Genotyping was done using GATK best practices with their build 38 resource bundle. The variants were lifted to hg19 using Picard (http://broadinstitute.github.io/picard/) and sites with >3 % missingness or mendelian discordance >5 % based on the large number of trios and duos present in the data set were removed. We phased the remaining sequencing data from the 447 Greenlanders using ShapeIt2 [22], using available duo/trio information. These phased data were then combined with 1000 genomes data for 99 CEU, 91 GBR, 103 CHB, 105 CHS, and 93 CDX to form the reference panel. For the 1000 genomes data, we included sites that overlapped with the whole genome data or had a minor allele frequency (MAF) > 1 %.

Using the constructed reference panel and duo/trio family information we then phased the MegaChip data for sites with more than five minor alleles for the 4182 individuals without WGS data with ShapIt2. The imputation was performed using IMPUTE2 [23]. The recombination map for the reference variants was inferred with linear interpolation using the hg19 genomic map from IMPUTE2 as a template, and an effective population size of 1,500 [24]. The imputation generated genotype data with an info score (*r*^2^) above 0.8 for 12,270,968 variants. Of these, we removed 16 tri-allelic variants on chromosome 12 and applied a MAF filter of 0.5%, resulting in a data set of 10,265,398 variants with a mean info score of >0.98, which we included in the analyses.

### Statistical analysis

#### Analyses of the Greenlandic data

To perform association analyses, we used a linear mixed model implemented in the software GEMMA [25] to account for the relatedness and population structure. For each phenotype, we included all individuals across the three cohorts with information about that phenotype, and the general relatedness matrix used as additional input in GEMMA was estimated from the same individuals using only SNPs with MAF above 5% and at most 1% missingness. We assumed an additive effect and included sex, age, cohort, and whether an individual had imputed or genotype data as covariates. Lipid levels were quantile transformed to a standard normal distribution within each sex before association analyses to obtain p-values and effect size estimates. In GWAS analyses, we applied a significance threshold of *p* < 5 × 10^−8^. QQ plots showed no indications of inflation due to confounding in analyses of the six lipid traits (Lambda values range from 0.99 to 1.02; Supplementary Fig. 1).

We estimated the partial variance explained (PVE) for the identified variants in the Greenlanders and for the variants reported in Europeans [4], using the formula [26]:

$${{{{{\rm{PVE}}}}}}\propto \frac{{\hat{\beta }}^{2}\cdot 2p(1-p)}{{\hat{\beta }}^{2}\cdot 2p\left(1-p\right)+{\left({{{{{\rm{SE}}}}}}\left(\hat{\beta }\right)\right)}^{2}N\cdot 2p(1-p)}$$

where *p* is the allele frequency, *N* is the number of individuals, *β* is the effects size, and SE is the standard error of the *β*.

CVD risk was assessed with Cox regression on the number of years lived until the first CVD event (survival time) using the R-package *survival* [27]. These analyses were adjusted for sex, age, and the top 10 principal components (PC) to correct for the population structure. Years until an event was calculated as the years from birth until an event or end of follow-up (2018).

#### Analyses of UK Biobank data

Variants of interest were analysed in self-reported white individuals from the UK Biobank version 3 [28] (UK Biobank project ID: 32683). LDL-cholesterol (Field ID: 30780 ‘LDL direct’), total cholesterol (Field ID: 30690 ‘Cholesterol’), triglycerides (Fiels ID: 30870 ‘Triglycerides’), HDL-cholesterol (Field ID: 30760 ‘HDL-cholesterol’), ApoA (Field ID: 30630 ‘Apolipoprotein A’), and ApoB (Field ID: 30640 ‘Apolipoprotein B’) were rank-based inverse normal transformed separately for each sex and analysed as continuous traits using Regenie [29] (v3.2.8) controlling for population structure and relatedness. Per the Regenie recommendations for UK Biobank analysis, we used the genotyped variants with MAF > 1%, MAC > 100, missing<10%, and HWE *p* value > 1 × 10–15, filtered using plink [30]. Both step 1 and step 2 were carried out with block size 1000 and otherwise default parameters. CVD risk was assessed using Cox regression, defining CVD events by the diagnosis codes in supplementary Tables 1,2 extracted using the field IDs: 41270 (ICD10), 41271 (ICD9), 41272 (OPCS), 20002 (self-reported), and 20004 (self-reported operations) and their related data fields for date of diagnosis/operation. All analyses were adjusted for birth year and month, sex, and the first 10 PCs, and conditioned on the *PCSK9* loss-of-function variant (rs11591147) to obtain the independent effect estimates for the *PCSK9* rs12117661 variant.

### Genetic risk scores

For the Greenlandic individuals, genetic risk scores (GRS) were calculated for each of the six lipid traits, by including variants identified by the GWAS (*p* < 5 × 10^−8^) and using the estimated effect size from the GWAS as weight. Because of our low sample size, our effects size estimates can suffer from winner’s curse [31], and estimating GRS performance on the same data where the effect sizes were estimated, could also overestimate the effect. To avoid this we did 10-fold cross-validation, dividing all the individuals randomly into 10 groups of equal size and estimating the GRS of the individuals in each group by using variants and effect sizes from a GWAS performed on the remaining 9 groups combined. Each of these GWAS was performed as the main lipid GWAS performed in this study. To avoid including multiple variants that associate due to LD clumping is often performed. However, because the Greenlanders are admixed, the LD measures will be inflated and will not accurately reflect LD in the ancestral (unadmixed) population. Therefore, we used a more stringent strategy where we sorted all the variants by p-value, kept the top variant, and discarded all other variants in a +/- 5 Mb region around it. We repeated this procedure until there were no more variants left with *p* < 5 × 10^−8^. In all 10 cross validations, there were 11 or fewer variants used. We calculated the variance explained by each GRS by the partial r^2^ of the GRS in a model adjusted for age, sex, and the first 10 PCs using quantile transformed lipid levels.

### In-silico variant analyses

The Ensembl database [32] and Encode 3 data [33] were queried to assess the co-localisation of variants with regulatory elements, such as transcription factor binding sites, promoter regions, and regions of DNase hypersensitivity. Moreover, RNA expression data from 48 tissues (with >70 samples, range: 80–399) were queried through the GTEx Portal to assess possible effects of the genetic variants on the expression of nearby genes [34].

## Results

**Fig. 1:**
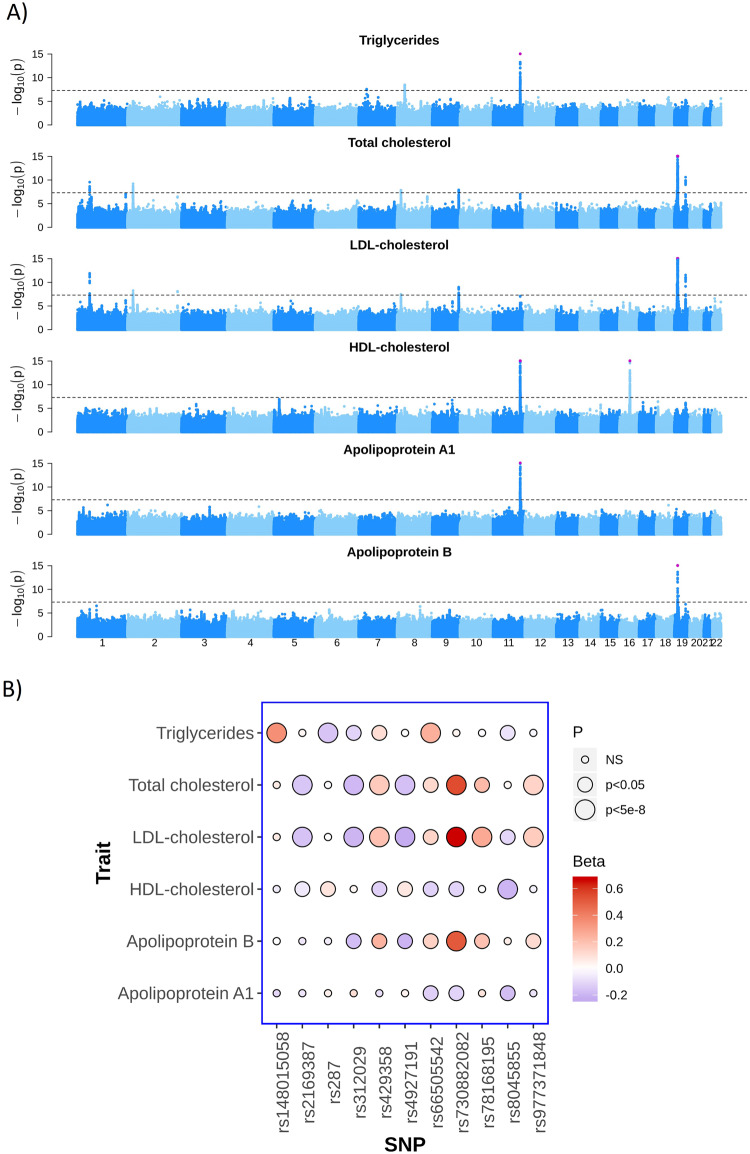
Genome-wide association studies of six lipid traits. **A** Manhattan plot for each trait. Association signals have been truncated at −log(*p*) = 15 indicated by pink dots. The dashed line indicates genome-wide significance (*p* < 5 × 10^−8^). **B** Effect size estimates (beta) and significance level (*p*) for all combinations of the six lipid traits and the lead variants from the 11 genome-wide significant loci identified in the GWAS. ApoA1 apolipoprotein A1, apoB apolipoprotein B. HDL-C HDL-cholesterol, LDL-C, LDL-cholesterol, TC total cholesterol, TG, triglycerides.

We identified 11 loci that were genome-wide significantly (*p* < 5 × 10^−8^) associated with at least one of the six lipid traits (Fig. 1A, Table 1) in up to 4473 Greenlanders (Supplementary Table 3 shows participant characteristics). Lead variants from ten of the 11 loci were at least nominally associated with more than one of the lipid traits at *p* < 0.05 (Fig. 1B and Supplementary Table 4).

**Table 1:** Lead variants of the 11 genome-wide significant loci with position on hg19.

Variant	Chr:pos (effect allele)	Variant type (Nearby genes)	MAF (%) GR	MAF (%) EU	Lipid trait	*N*	*β* (SE)	*β*_SD_ (SE)	*p* value	PVE (%)
rs78168195	2:228480653 (G)	Intron (C2orf83)	6.1	8.1	LDL-C (mmol/L)	3809	0.33 (0.05)	0.27 (0.05)	7.9 × 10^−9^	0.87
rs148015058	7:25766657 (T)	Intergenic (*NFE2L3*, *NPVF*)	3.9	14.4	TG (mmol/L)	3970	0.19 (0.04)	0.35 (0.06)	2.6 × 10^−8^	0.77
rs4927191	1:55491702 (C)	Intergenic (*PCSK9*)	16.4	24.3	LDL-C (mmol/L)	3809	−0.23 (0.03)	−0.23 (0.03)	1.2 × 10^−12^	1.29
rs312029	2:21461730 (C)	Intergenic (*TDRD15*, *APOB*)	14.0	12.9	TC (mmol/L)	4341	−0.23 (0.04)	−0.19 (0.03)	6.2 × 10^−10^	0.87
rs2169387 (38)	8:9181395 (A)	Intergenic (*PPP1R3B*)	18.6	9.7	TC (mmol/L)	4342	−0.18 (0.03)	−0.15 (0.03)	1.5 × 10^−8^	0.74
rs287 (38)	8:19815556 (G)	Intron (*LPL*)	29.3	25.2	Triglycerides (mmol/L)	3970	−0.11 (0.02)	−0.16 (0.03)	3.3 × 10^−9^	0.90
rs2519093	9:136141870 (T)	Intron (*ABO*)	25.6	19.8	LDL-C (mmol/L)	3809	0.19 (0.03)	0.16 (0.03)	1.0 × 10^−9^	0.95
rs66505542	11:116623213 (TA)	Intron (*BUD13*)	28.9	15.1	TG (mmol/L)	3970	0.16 (0.02)	0.25 (0.03)	1.3 × 10^−22^	2.46
rs8045855	16:57000696 (A)	Intron (*CETP*)	48.9	18.4	HDL-C (mmol/L)	4473	−0.10 (0.01)	−0.20 (0.02)	6.7 × 10^−20^	1.81
rs730882082	19:11215991 (A)	Missense (*LDLR*)	15.8	0	LDL-C (mmol/L)	3809	0.75 (0.03)	0.66 (0.03)	7.2 × 10^−99^	10.90
rs429358	19:45411941 (C)	Missense (*APOE*)	21.3	14.9	LDL-C (mmol/L)	3809	0.20 (0.03)	0.20 (0.03)	2.8 × 10^−12^	1.26

Effect sizes and corresponding standard errors (SE) are shown for the lipid trait with the strongest association as untransformed (β) and quantile transformed (β_SD_) values. P-values were calculated based on the transformed trait values. Rs78168195 and rs148015058 are potentially novel variants and the remaining loci have been associated with lipid levels previously [13, 38]. Minor allele frequencies (MAF) are shown for Greenlanders (GR) and for Europeans (EU). The European frequencies were obtained for the non-Finnish Europeans from the GnomAD data set v.2 (https://gnomad.broadinstitute.org/).

*PVE* partial variance explained, *HDL-C* HDL-cholesterol, *LDL-C* LDL-cholesterol, *TC* total cholesterol, *TG* triglycerides.

Two of the 11 loci, on chromosome 2 (rs78168195) and chromosome 7 (rs148015058), were potentially novel (Table 1 and Supplementary Fig. 2A, B). The locus on chromosome 2 was primarily associated with higher levels of LDL-cholesterol, and the locus on chromosome 7 was associated with higher levels of triglycerides (Table 1 and Supplementary Table 4). The lead variants from the two loci were more common in Europeans than in Greenlanders (Table 1) and, therefore, if these signals were true, they should be genome-wide significantly associated with the same lipid traits in Europeans. In 1,230,380 and 1,115,719 Europeans, respectively, rs78168195 showed no association with LDL-cholesterol levels (*p* = 0.585), and rs148015058 showed no association with triglyceride levels (*p* = 0.329) [4]. Based on these findings, these signals were unlikely to be true signals.

The lead variants from the remaining nine of the 11 loci were located in previously reported lipid loci (Table 1). However, in the locus on chromosome 1, near *PCSK9*, we identified a signal which was not explained by previously reported lead variants. We explored this signal in more detail.

### A possible causal variant near *PCSK9*

The minor allele of the lead variant (rs4927191) in the locus near *PCSK9* on chromosome 1 was associated with lower levels of LDL-cholesterol (Table 1), total cholesterol (−0.17 (0.03), *p* = 2.3 × 10^−9^), and ApoB (−0.20 (0.05), *p* = 1.5 × 10^−4^), as well as higher levels of HDL-cholesterol (0.08 (0.03), *p* = 0.0082) (Supplementary Table 4).

**Fig. 2:**
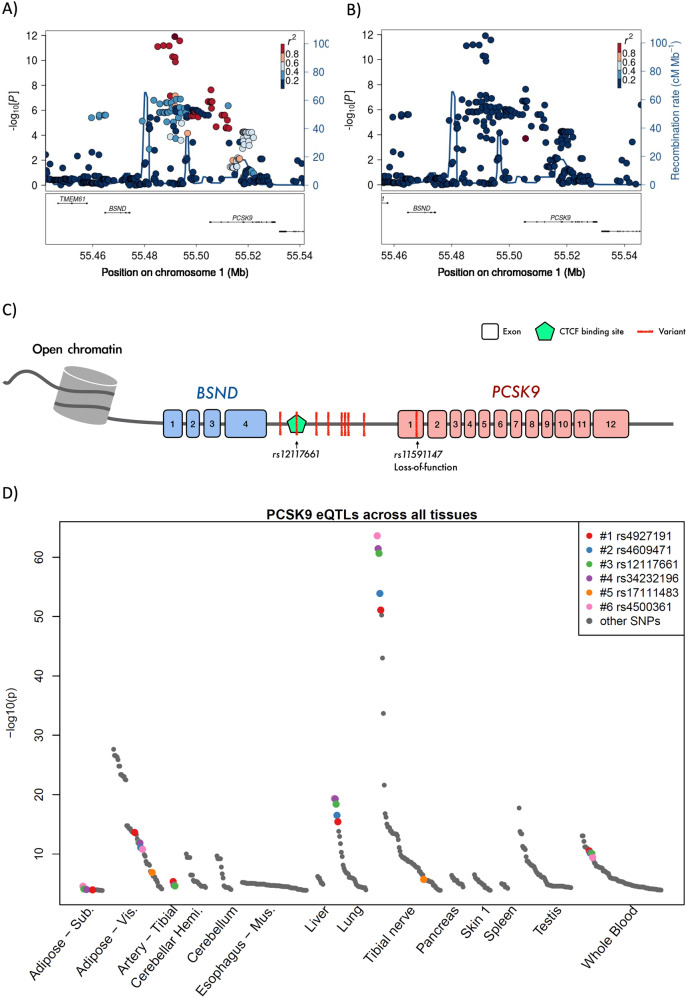
Association signal near *PCSK9*. **A** Locus zoom of the LDL-cholesterol association signal at chromosome 1. The colour of the dots indicates the linkage equilibrium in the Inuit ancestry only with the lead variant rs4927191 (dark red dot). **B** The LDL-cholesterol association signal conditioned on the *PCSK9* loss-of-function variant (rs11591147). **C** Schematic representation of the *PCSK9* locus. **D** eQTL signals for *PCSK9* across tissues from GTEx with *p* < 2.0 × 10^−4^. The top 6 variants from the LDL-cholesterol-association signal near *PCSK9* are highlighted with colours Adipose-Sub, subcutaneous adipose tissue; Adipose Vis, visceral adipose tissue; Cerebellar Hemi, Cerebellar Hemisphere; Oesophagus - Mus, Oesophagus - Muscularis; Skin 1, not sun-exposed skin (Suprapubic). Data were obtained from the GTEx Portal [34].

Importantly, these associations were independent of the well-established, causal *PCSK9* LoF variant (rs11591147); conditioning on this LoF variant did not attenuate our signal for any of the lipid traits (Fig. 2A, B), and the LoF variant had an estimated allele frequency of 0% in the Inuit ancestry and 0.6% in the Greenlandic population. Also, another coding *PCSK9* variant (rs143117125), suggested to be causal in Europeans, was too rare to be found in our whole genome sequencing data of 447 individuals, and could, therefore, not explain the observed associations either.

Our independent *PCSK9*-association signal comprised several variants in high LD. Particularly eight variants (#1-#8), all with pairwise r2 values above 0.8, separated from the rest of the association signal with p-values below 10^−9^ for association with the primary trait, LDL-cholesterol in the Greenlanders (Fig. 2A and Supplementary Table 5). These variants were also strongly associated with LDL-cholesterol and total cholesterol in UK biobank Europeans, even when conditioning on the PCSK9 LoF variant (Supplementary Table 5). These eight top variants (#1-#8) were all intergenic and located between *BSND* and *PCSK9* (Fig. 2C). In the GTEx Portal, we could assess if six of the top variants (#1-#6) were associated with *PCSK9* expression across 48 tissues, rs200159426 (#7) and rs187607506 (#8) were not available. Interestingly, five of the top variants (rs4927191 (#1), rs4609471 (#2), rs12117661 (#3), rs34232196 (#4), and rs4500361 (#6)) were the five variants most significantly associated with expression of *PCSK9* across tissues in GTEx (Fig. 2D, Supplementary Table 6), indicating that the causal variant could be one of these five variants. *PCSK9* expression was significantly affected in the tibial nerve, visceral and subcutaneous adipose tissue, lung, whole blood, and the tibial artery (Supplementary Table 6). Next, we queried the Ensembl database and Encode 3 data, and found that rs12117661 (#3) was the only of the eight potential causal variants, which colocalized with in a predicted regulatory region, namely a CTCF binding site (ENSR00000251959) in a region with open chromatin (Fig. 2C).

**Fig. 3:**
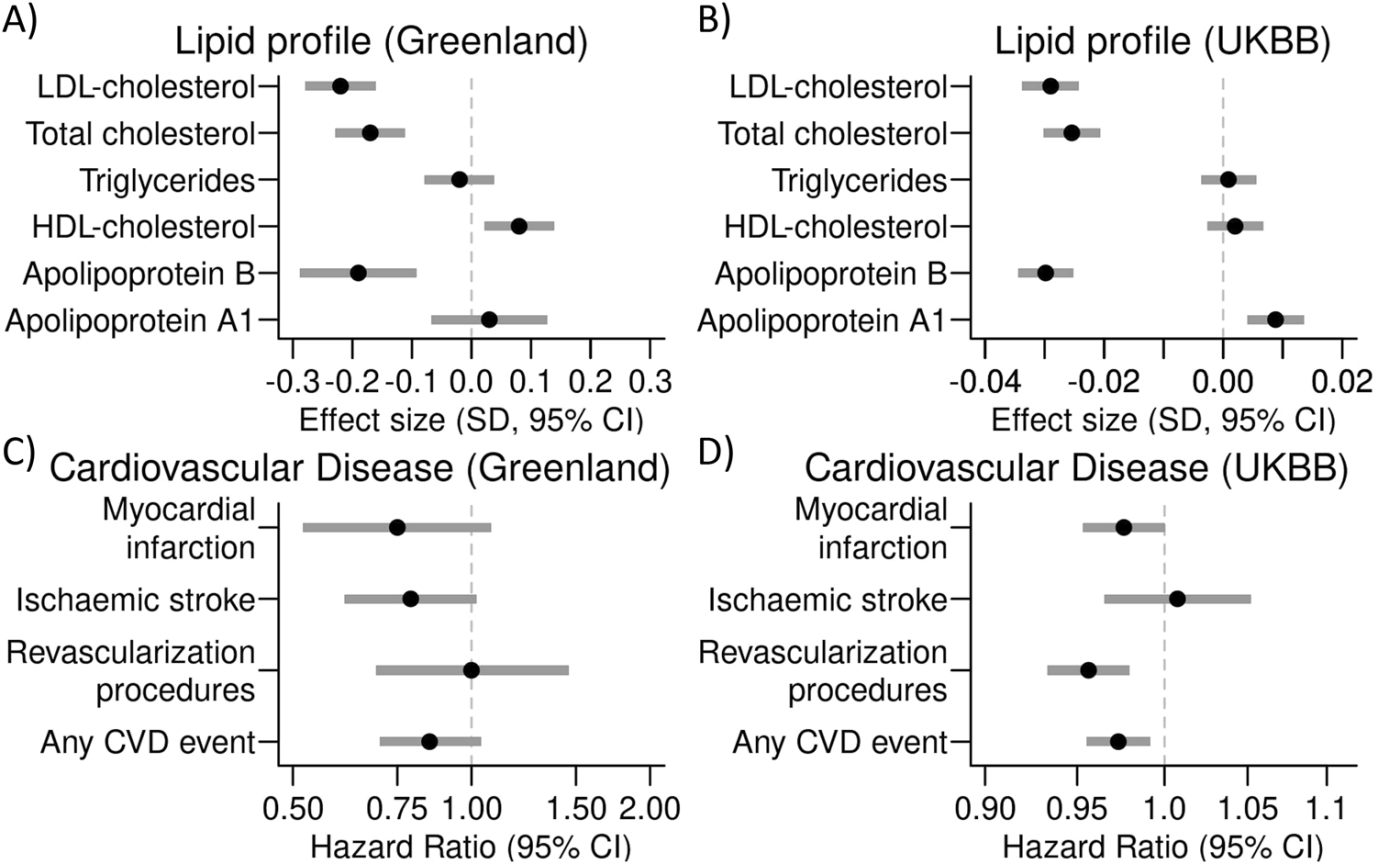
Association analyses of the potential causal variant near *PCSK9*. Association results for rs12117661 and Circulating lipid levels in (**A**) Greenlanders and (**B**) Europeans from the UK Biobank, as well as cardiovascular disease outcomes in (**C**) Greenlanders and (**D**) Europeans from the UK Biobank. Hazard ratios and 95% confidence intervals were estimated with a Cox regression model adjusted for age, sex, and PC1-10.

The *PCSK9* rs12117661 (#3) variant was the strongest candidate to be the causal variant, and we, therefore, assessed associations with additional cardiometabolic traits (results for the other seven *PCSK9* top variants are presented in Supplementary Table 5 and Supplementary Table 7). The minor allele of rs12117661 (#3) was associated with lower levels of LDL-cholesterol, total cholesterol, and ApoB, as well as higher levels of HDL-cholesterol (Fig. 3A and Supplementary Table 8). We observed no associations between rs12117661 (#3) and measures of glucose homeostasis, blood pressure, or measures of body composition (Supplementary Table 8). In data from 3,716 Greenlandic individuals with a median time lived until first event (or end of follow-up) of 56.2 years, rs12117661 (#3) was not significantly associated with risk of ischaemic stroke, myocardial infarction, revascularization procedures, or a combined measure of CVD events (Fig. 3D and Supplementary Table 7). However, the point estimates indicated a potential markedly lower risk of CVD events. To substantiate these findings, we tested for similar association results in the much larger European UK Biobank cohort. Here, the minor allele of rs12117661 (#3) had a frequency of 24.6% and was associated with lower levels of LDL-cholesterol, total cholesterol, and ApoB, even when conditioning on the *PCSK9* LoF variant (Fig. 3B, Supplementary Table 5), and with a lower risk of revascularization procedures and any CVD event (Fig. 3D, Supplementary Table 7).

### Impact of the identified variants

We also assessed the impact on CVD of the lead variants from the remaining 10 loci that were significantly associated with one of the six lipid traits in the initial GWAS analyses. The derived allele of the *LDLR* rs730882082 variant was associated with a higher risk of revascularization procedures and any CVD event (Supplementary Table 9), in line with what has been shown previously in this study population [14]. Furthermore, the lead variant from the *APOB* association signal on chromosome 2 (rs312029) was nominally associated with a higher risk of revascularization procedures (Supplementary Table 9).

We also assessed how much of the trait variance was explained by the lead variant from each of the 11 loci, as well as the potential novel causal *PCSK9* variant. This varied between 0.74% and 10.9% for the lead variants (Table 1), and the *PCSK9* rs12117661 variant explained 1.25% of the LDL-cholesterol variance (Supplementary Table 5). Compared to variants identified in European GWAS of the same traits, our lead variants were relatively common and explained a larger proportion of the variance (Supplementary Fig. 3), suggesting that the genetic architecture of these traits in Greenlanders comprises a lower number of variants of either high frequency, high effect, or both.

**Fig. 4:**
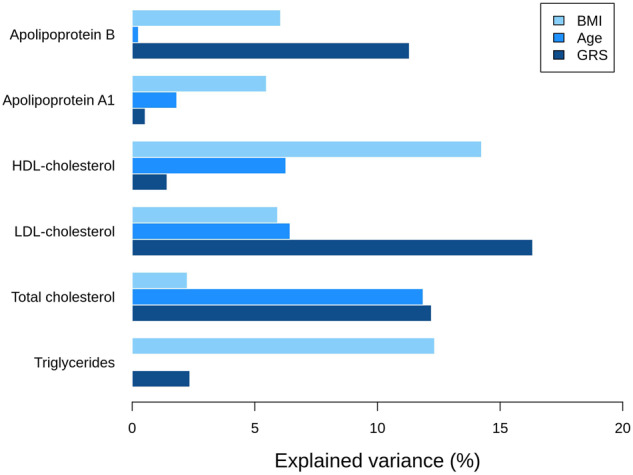
Partial variance explained by the genetic risk scores (GRS), age, and BMI. Explained variance was estimated for each lipid trait as the partial r^2^ in a model including all variables and adjusted for age, BMI, and principal components 1–10.

Finally, to further investigate the combined impact of the lead variants for each of the six lipid traits, from the 11 genome-wide significant loci, we generated a separate GRS for each trait. There was a correlation between the GRS for LDL-cholesterol, total cholesterol, and ApoB, as well as between the GRS for HDL-cholesterol and ApoA (Supplementary Fig. 4). Interestingly, the GRS explained from 0.53% for ApoA1 to as much as 16.3% for LDL-cholesterol when adjusting for BMI, age, and PC1-10 (Fig. 4, Supplementary Table 10) and from 0.73% for apoA1 to 13.7% for LDL-cholesterol when only adjusting for PC1-10 (Supplementary Table 10). For triglycerides, total cholesterol, LDL-cholesterol, and ApoB, the GRS explained more of the variance than did age, and for total cholesterol, LDL-cholesterol, and ApoB, the GRS also explained more than BMI (Fig. 4, Supplementary Table 10). Notably, the *LDLR* rs730882082 variant explained far more of the variance in LDL-cholesterol, total cholesterol, and ApoB than the remaining variants in the GRS, namely 10.9%, 7.6%, and 6.18% (Supplementary Table 4).

## Discussion

In our GWAS analyses of six lipid traits in Greenlanders, we identified 11 distinct loci harbouring variants that were genome-wide significantly associated with at least one of these traits. Nine of the 11 signals mapped to loci previously reported to be associated with circulating lipid levels [4, 5]. The remaining two loci were potentially novel, however, unlikely to be true signals. Notably, we identified a potential novel independent causal variant (rs12117661) near *PCSK9* on chromosome 1.

The strongest association across the lipid traits was observed for the *LDLR* rs730882082 variant and LDL-cholesterol, with an effect of 0.66 SD, corresponding to 0.75 mmol/L, per effect allele, as previously observed [14]. For the rest of the genome-wide significant variants across traits, the effect sizes ranged from −0.20 SD to 0.35 SD, these effect sizes might, however, be slightly overestimated with an effective sample size of a maximum of 4,473 and the impact of winner’s curse [31].

Importantly, the *PCSK9* LoF variant (rs11591147), which is strongly associated with LDL-cholesterol in Europeans, and has been hypothesised to be the causal variant in this locus [35, 36], was absent from the Inuit ancestry and had a low MAF in the general Greenlandic population. Furthermore, the LoF variant was not associated with levels of circulating lipids in our Greenlandic study population, and conditioning on this variant did not attenuate the association signal for our lead variant (rs12117661) in the *PCSK9* signal. Therefore, our signal near *PCSK9* was independent of the LoF variant. A lipid-associated *PCSK9* signal independent of the LoF variant has previously been reported in Europeans, and it was suggested that the rare missense variant rs143117125 (p.N157K) was the causal variant in this signal [10]. This variant was too rare to be detected in the Greenlandic whole genome sequencing data and could, therefore, not explain our association signal either.

We identified the rs12117661 variant as a potential causal variant in the *PCSK9* association signal. This variant was among the variants showing the strongest association with lower levels of LDL-cholesterol and total cholesterol in the Greenlanders. Analyses of data from the UK Biobank showed strong associations as well, even when conditioning on the *PCSK9* LoF variant, indicating that this rs12117661 signal is shared between Europeans and Greenlanders. Of note, the observed effect sizes for rs12117661 in Europeans were markedly lower than in the Greenlandic cohort. This could potentially indicate the presence of another causal variant in the Greenlanders, which could not be detected with the current high depth short read sequencing data.

In Greenlanders, rs12117661 also showed a non-significant association with a lower risk of peripheral artery disease, and in Europeans, it was very significantly associated with a lower risk of revascularization procedures and any CVD event, even when conditioning on the *PCSK9* LoF variant. In silico analyses showed that the rs12117661 variant colocalized with a predicted transcription factor binding site, indicating that this variant might affect the transcript of nearby genes, including *PCSK9*. Moreover, rs12117661 was strongly associated with lower *PCSK9* expression levels in multiple tissues in European data from the GTEx portal [34]. Lower *PCSK9* expression is consistent with the observed association with lower levels of circulating LDL-cholesterol and total cholesterol, as lower PCSK9 activity will increase LDL-receptor recycling and thereby increase hepatic LDL-cholesterol uptake and degradation [37]. Further studies of gene expression are needed to verify that the effect is similar in Greenlanders and to identify the specific tissues where the variant exerts its effect to cause lower lipid levels. Gene expression data in the Greenlanders across tissues would also allow for colocalization analyses, which might further support the functional interpretation.

The *PCSK9* rs12117661 variant had an effect size for LDL-cholesterol of −0.22 (0.03) SD and explained 1.3% of the variance in LDL-cholesterol levels in Greenlanders. Combined with an effect allele frequency of 15.6%, the impact of this variant is relatively large in the Greenlandic population. In comparison, the effect size of the *PCSK9* LoF variant (rs11591147) on LDL-cholesterol of up to −0.53 SD in Europeans is larger [38, 39], but the LoF variant only has a frequency of 1–2% in Europeans [10, 38, 39], and therefore only explains 0.52% of the variance in LDL-cholesterol in Europeans [4]. The population-level impact is, therefore, much larger for the identified *PCSK9* rs12117661 variant in Greenlanders, than for the LoF variant in Europeans.

The combination of a relatively large effect size and high frequency makes the *PCSK9* rs12117661 variant useful for assessing the long-term effects of using PCSK9 inhibitors to treat hypercholesterolaemia. This is of great interest since most trials so far have only assessed the effects of the *PCSK9* inhibitors with no more than 3 years of follow-up [40, 41]. Assessing possible long-term effects is particularly relevant, as there are concerns about PCSK9 inhibitors exhibiting adverse neurocognitive effects as well as increasing the risk of type 2 diabetes and cancer [41–43]. The link to type 2 diabetes is further supported by studies of the *PCSK9* rs11591147 LoF variant [44]. Contrary to these findings, we did not observe any indications of adverse glucose homeostasis in carriers of the rs12117661 variant in the Greenlandic data. This is consistent with findings from the UK Biobank [45] and the FinnGen study (http://r7.finngen.fi/), where we observed no association between the rs12117661 variant and type 2 diabetes. Moreover, in the same datasets, no association with any type of cancer was observed. Instead, we showed that the rs12117661 variant was associated with a beneficial effect on CVD outcomes in Europeans from the UK Biobank, and the variant was associated with lower risk of similar outcomes in the FinnGen study (http://r7.finngen.fi/). We were unable to show any significant effect of the rs12117661 variant on CVD outcomes in the Greenlanders, likely due to the low number of CVD events. Additional analyses in larger study populations are needed to address these adverse and beneficial effects of the variant further.

### Genetic architecture of lipid traits in Greenlanders

We found that the Greenlandic population has a unique genetic architecture of circulating lipid levels comprising relatively common variants with large effect sizes explaining more variance than variants in Europeans [4]. Combined, variants from the 11 genome-wide significant signals explained a large amount of the variance in Greenlanders, particularly for LDL-cholesterol with PVE of 16.3%. This was mainly driven by the *LDLR* rs730882082 variant, which has previously been found to have a very large effect on LDL- and total cholesterol in Greenlanders [14]. In Europeans, up to 22% of the variance in LDL-cholesterol has been explained by GRS, but these scores comprised up to 2 million variants [4–7]. Taken together, the genetic architecture of lipid traits in Greenlanders is similar to what has been reported for other metabolic traits, where few variants explain a large proportion of the variance [11, 12].

In conclusion, we identified 11 independent loci associated with levels of at least one of six lipid traits in Greenlanders, including an independent variant associated with LDL- and total cholesterol levels through regulation of *PCSK9* expression. The suggested causal variant (rs12117661) in the *PCSK9* locus colocalized with a predicted regulatory site and was strongly associated with lipid levels in Greenlanders, and lower expression of *PCSK9* and lower risk of certain CVD outcomes in Europeans. Our results revealed a unique genetic architecture of lipid traits among Greenlanders, where few variants explained a large amount of the trait variance.

### Supplementary information

Supplementary material

## Data Availability

The data generated for this paper can be found within the published paper and its supplementary file. The accession numbers for the genotype data are EGAD00010001427, EGAD00010001428, and EGAD00010002057 (https://ega-archive.org/datasets).
