# Supplementary material for: GWAS of lipids in Greenlanders finds association signals shared with Europeans and reveals an independent PCSK9 association signal
Source: Eur J Hum Genet. 2023 Oct 30;32(2):215–23. doi: 10.1038/s41431-023-01485-8 (PMC10853193; doi:10.1038/s41431-023-01485-8)
Supplement: Supplementary file 1 — Supplementary material [file 41431_2023_1485_MOESM1_ESM.docx]

Supplemental material

GWAS of lipids in Greenlanders finds association signals shared with Europeans and reveals an independent *PCSK9* association signal

**Supplementary Figures**


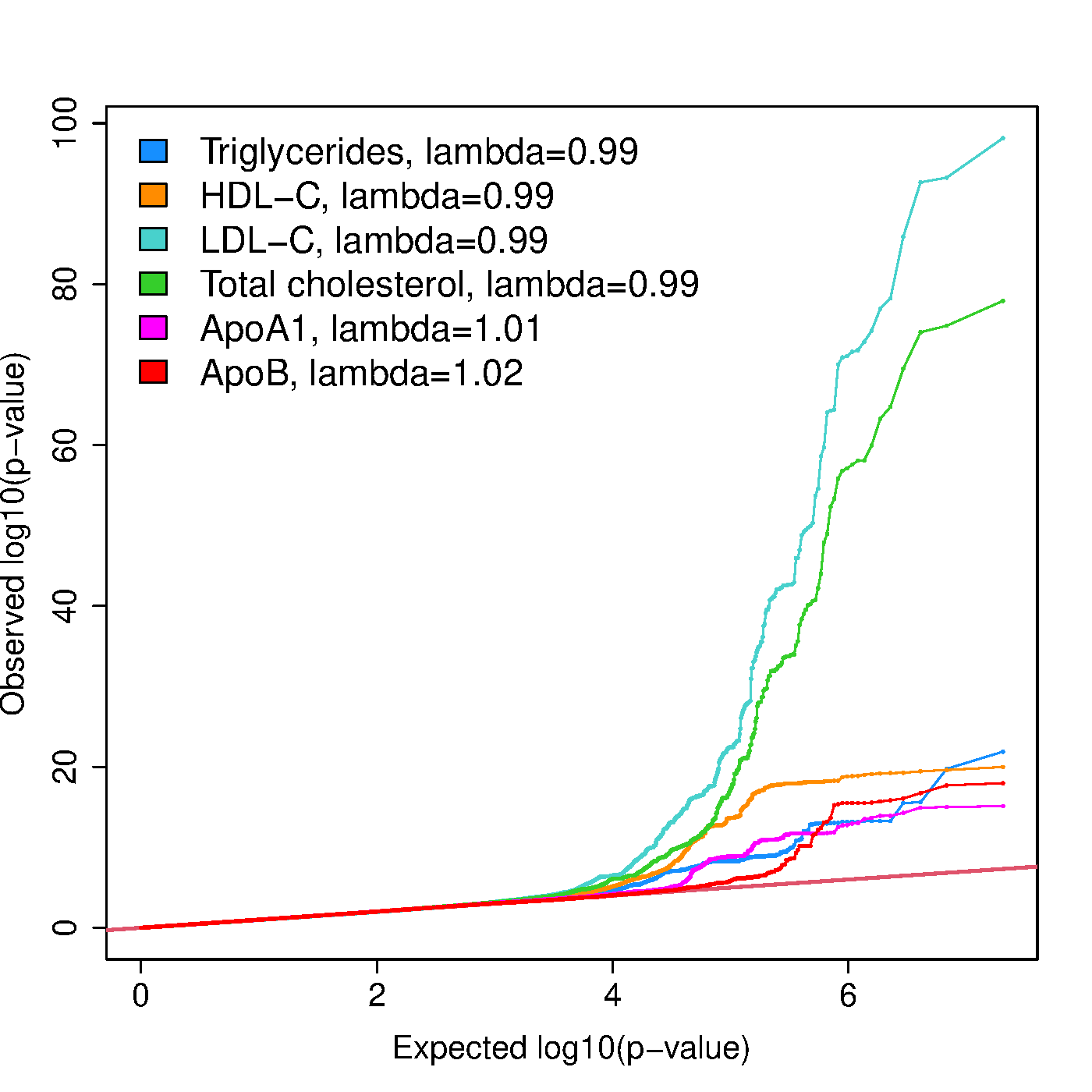


**Supplementary Figure 1.** QQ-plots for each of the genome-wide association analyses of the six lipid traits and the corresponding lambda values.

**A)   B)**


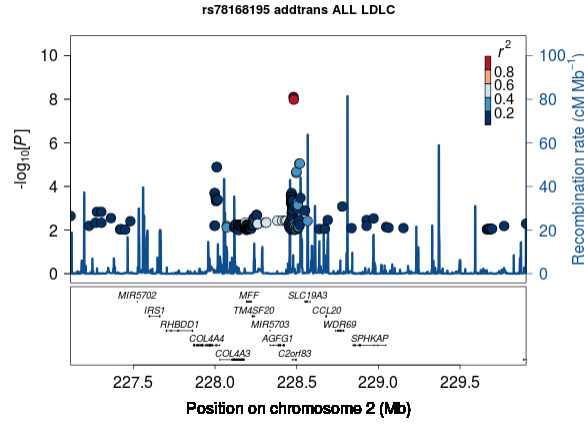
  
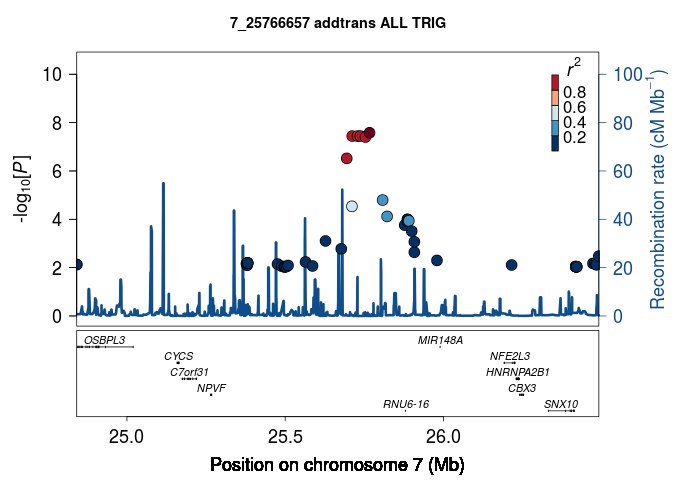


**Supplementary Figure 2.** Locus zoom of the potentially novel association signals for A) LDL-cholesterol at chromosome 2 (lead variant rs78168195), and for B) triglycerides at chromosome 7 (lead variant rs148015058).


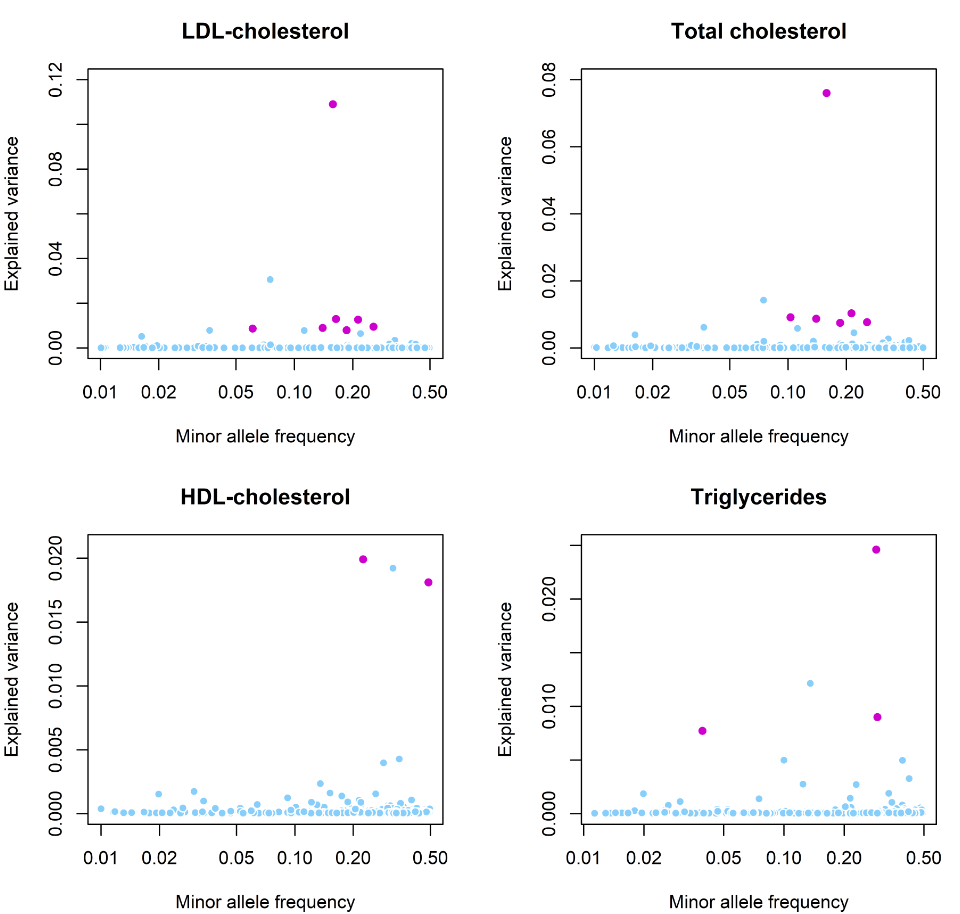
**Supplementary Figure 3.** Explained variance plotted against allele frequencies for variants with MAF>1% associated with each of the six lipid traits at p<5x10^-8^. Lead variants of the association signals identified in Greenlanders (pink), and variants associated with the lipid traits in Europeans (blue) (1).


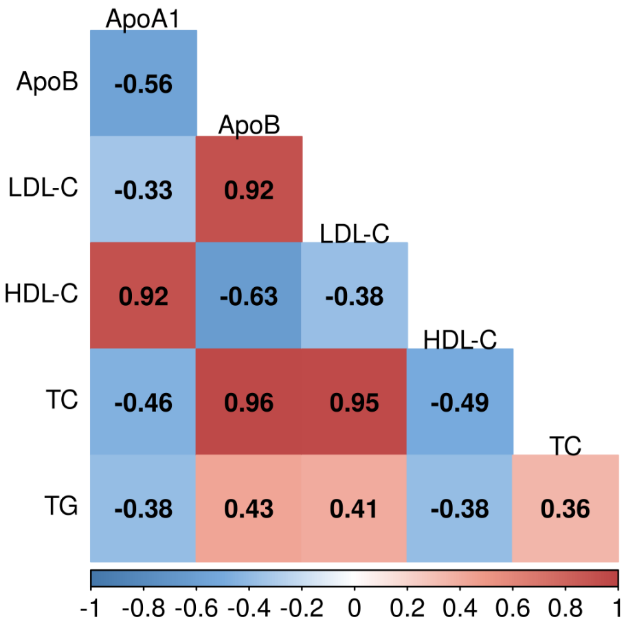


**Supplementary Figure 4.** Spearman’s correlation coefficients between the genetic risk scores for each of the six lipid traits. ApoA1, apolipoprotein A1; apoB, apolipoprotein B; HDL-C, HDL-cholesterol; LDL-C, LDL-cholesterol, TC, total cholesterol; TG, triglycerides.

**Supplementary Tables**

|  | **ICD8**  (Greenland) | **ICD9**  (UKBB) | **ICD10**  (Greenland+UKBB) | **ICPC2**  (Greenland) | **Self-reported**  (UKBB) |
| --- | --- | --- | --- | --- | --- |
| **Myocardial Infarction** | 41009, 41099, 41109, 41199, 41209, 41299 | 410, 411, 412, 42979 | I21, I22, I23, I241, I252 | K74, K75, K76 | 1075 |
| **Ischaemic stroke** | 43309, 43399, 43409, 43499, 43601, 43609, 43690, 43699 | 434, 436 | I63, I64 | K89, K90, K91 | 1583 |

**Supplementary Table 1.** The definition of cardiovascular disease outcomes is based on diagnosis codes according to previously reported categories (2). ICD, International Statistical Classification of Diseases and Related Health Problems; ICPC, International Classification of Primary Care; Self-reported: UK Biobank specific codes for self-reported diseases.

| **The Classification of Operations and Treatments in Greenland**  (Greenland) | **NOMESCO Classification of Surgical Procedures**  (Greenland) | **OPCS-4**  (UKBB) | **Baseline medical history**  (UKBB) |
| --- | --- | --- | --- |
| 30009, 30019, 30029, 30039, 30049, 30059, 30069, 30079, 30089, 30099, 30109, 30119, 30120, 30129, 30139, 30149, 30159, 30169, 30179, 30189, 30199, 30200, 30240, 30241, 30245, 30280, 86011, 86012, 86013, 86020, 86111, 86112, 86113, 86211, 86212, 86213, 86311, 86312, 86313, 86611, 86612, 86613, 86711, 86712, 86713, 86811, 86812, 86813, 86911, 86912, 86913, 86920, 87011, 87012, 87013 | KFNC, KFNC, KFNC, KFNC, KFNC, KFNA, KFNW96, KFNG, KNFG, KFNG12, KNFG, KNFG, KPA | K40, K41, K42, K43, K44, K45, K46, K49, K50, K553, K75, K76, L29, L303, L311, L313, L314, L318, L319 | 1069, 1070, 1095, 1105 |

**Supplementary Table 2.** The Classification of Operations and Treatments and The Nordic Medico-Statistical Committee (NOMESCO) Classification of Surgical Procedures were used to classify revascularization procedures for Greenlanders, and Classification of Interventions and Procedures (OPCS-4) and baseline medical history were used to classify revascularization procedures for Europeans from the UK Biobank.

| **Phenotype** | **All** | | **Males** | | **Females** | |
| --- | --- | --- | --- | --- | --- | --- |
|  | Median (25-75th percentile) | N | Median (25-75th percentile) | N | Median (25-75th percentile) | N |
| Age (years) | 43 (33-54) | 4493 | 44 (34-55) | 1880 | 43 (33-53) | 2613 |
| LDL-cholesterol (mmol/L) | 3.6 (2.9-4.3) | 3808 | 3.6 (2.9-4.4) | 1658 | 3.5 (2.9-4.3) | 2150 |
| HDL-cholesterol (mmol/L) | 1.6 (1.3-1.9) | 4472 | 1.5 (1.2-1.89) | 1871 | 1.66 (1.4-2) | 2601 |
| Total cholesterol (mmol/L) | 5.8 (5.1-6.6) | 4341 | 5.8 (5.1-6.6) | 1811 | 5.8 (5.1-6.675) | 2530 |
| Triglycerides (mmol/L) | 1.0 (0.75-1.39) | 3969 | 0.98 (0.73-1.42) | 1737 | 1.02 (0.76-1.37) | 2232 |
| ApoA1 (g/L) | 1.71 (1.55-1.95) | 1196 | 1.66 (1.48-1.87) | 511 | 1.77 (1.61-1.98) | 685 |
| ApoB (g/L) | 0.89 (0.76-1.05) | 1196 | 0.89 (0.76-1.06) | 511 | 0.90 (0.77-1.04) | 685 |
| Use of lipid lowering drugs (%) | 3.0 | 2770 | 3.3 | 1227 | 2.8 | 1543 |
| HbA1c (%) | 5.8 (5.5-6.1) | 4446 | 5.8 (5.5-6.1) | 1869 | 5.7 (5.4-6) | 2577 |
| Fasting plasma glucose (mmol/L) | 5.6 (5.2-6) | 3556 | 5.7 (5.3-6.1) | 1530 | 5.5 (5.2-5.9) | 2026 |
| 2-h plasma glucose (mmol/L) | 5.4 (4.3-6.7) | 3321 | 5.0 (4.0-6.4) | 1438 | 5.7 (4.7-7.1) | 1883 |
| Fasting serum insulin (pmol/L) | 38 (25-56) | 3554 | 33 (22-50) | 1531 | 41 (28-60) | 2023 |
| 2-h serum insulin (pmol/L) | 108 (47-210) | 3322 | 61 (30-136) | 1439 | 146 (76-247) | 1883 |
| Use of glucose lowering drugs (%) | 1.4 | 2781 | 1.3 | 1232 | 1.4 | 1549 |
| Systolic blood pressure (mm Hg) | 76 (69-84) | 3983 | 78 (70-86) | 1694 | 74 (68-82) | 2289 |
| Diastolic blood pressure (mm Hg) | 122 (111-134) | 3983 | 127 (116-138) | 1694 | 119 (110-130) | 2289 |
| Use of antihypertensive drugs (%) | 10.2 | 2822.0 | 9.0 | 1241.0 | 11.1 | 1581 |
| BMI (kg/m^2^) | 25.3 (22.4-29.0) | 4446 | 25.0 (22.4-28.5) | 1858 | 25.5 (22.4-29.5) | 2588 |

**Supplementary Table 3**. Participants Characteristics.

| **Locus** | **Variant ID** | **Chr:pos**  **(effect allele)** | **type of variant (Nearest gene)** | **Lipid trait** | **N** | **β_SD_ (SE)** | **p-value** | **PVE (%)** |
| --- | --- | --- | --- | --- | --- | --- | --- | --- |
| 1 | rs78168195 | 2:228480653 (G) | Intron (C2orf83) | LDL-cholesterol | 3809 | 0.27 (0.05) | 7.9x10^-9^ | 0.87 |
|  |  |  |  | Total cholesterol | 4342 | 0.22 (0.04) | 3.4x10^-7^ | 0.59 |
|  |  |  |  | ApoB | 1197 | 0.20 (0.09) | 0.027 | 0.41 |
| 2 | rs148015058 | 7:25766657 (T) | Intergenic (*NPVF*) | Triglycerides | 3970 | 0.35 (0.06) | 2.6x10^-8^ | 0.77 |
| 3 | rs4927191 | 1:55491702 (C) | Intergenic (*PCSK9*) | LDL-cholesterol | 3809 | -0.23 (0.03) | 1.2x10^-12^ | 1.29 |
|  |  |  |  | Total cholesterol | 4342 | -0.17 (0.03) | 2.3x10^-9^ | 0.83 |
|  |  |  |  | ApoB | 1197 | -0.20 (0.05) | 1.5x10^-4^ | 1.17 |
|  |  |  |  | HDL-cholesterol | 4473 | 0.08 (0.03) | 0.0082 | 0.15 |
| 4 | rs312029 | 2:21461730 (C) | Intergenic (*APOB*) | Total cholesterol | 4341 | -0.19 (0.03) | 6.2x10^-10^ | 0.87 |
|  |  |  |  | LDL-cholesterol | 3808 | -0.20 (0.03) | 5.6x10^-9^ | 0.89 |
|  |  |  |  | Triglycerides | 3969 | -0.12 (0.03) | 5.5x10^-4^ | 0.30 |
|  |  |  |  | ApoB | 1196 | -0.18 (0.07) | 0.0060 | 0.62 |
| 5 | rs2169387 | 8:9181395 (A) | Intergenic (*PPP1R3B*) | Total cholesterol | 4342 | -0.15 (0.03) | 1.5x10^-8^ | 0.74 |
|  |  |  |  | LDL-cholesterol | 3809 | -0.17 (0.03) | 3.5x10^-8^ | 0.79 |
|  |  |  |  | HDL-cholesterol | 4473 | -0.06 (0.03) | 0.025 | 0.11 |
| 6 | rs287 | 8:19815556 (G) | Intron (*LPL*) | Triglycerides | 3970 | -0.16 (0.03) | 3.3x10^-9^ | 0.90 |
|  |  |  |  | HDL-cholesterol | 4473 | 0.08 (0.02) | 4.6x10^-4^ | 0.27 |
| 7 | rs2519093 | 9:136141870 (T) | Intron (*ABO*) | LDL-cholesterol | 3809 | 0.16 (0.03) | 1.0x10^-9^ | 0.95 |
|  |  |  |  | Total cholesterol | 4342 | 0.14 (0.02) | 1.2x10^-8^ | 0.77 |
|  |  |  |  | ApoB | 1197 | 0.12 (0.05) | 0.016 | 0.49 |
| 8 | rs66505542 | 11:116623213 (TA) | Intron (*BUD13*) | Triglycerides | 3970 | 0.25 (0.03) | 1.3x10^-22^ | 2.46 |
|  |  |  |  | LDL-cholesterol | 3809 | 0.14 (0.03) | 9.7x10^-8^ | 0.77 |
|  |  |  |  | Total cholesterol | 4342 | 0.12 (0.02) | 1.4x10^-7^ | 0.64 |
|  |  |  |  | HDL-cholesterol | 4473 | -0.12 (0.02) | 1.5x10^-7^ | 0.64 |
|  |  |  |  | ApoB | 1197 | 0.15 (0.05) | 0.0016 | 0.84 |
|  |  |  |  | ApoA1 | 1197 | -0.13 (0.05) | 0.0045 | 0.66 |
| 9 | rs8045855 | 16:57000696 (A) | Intron (*CETP*) | HDL-cholesterol | 4473 | -0.20 (0.02) | 6.7x10^-20^ | 1.81 |
|  |  |  |  | LDL-cholesterol | 3809 | -0.11 (0.02) | 6.8x10^-6^ | 0.54 |
|  |  |  |  | ApoA1 | 1197 | -0.18 (0.04) | 3.5x10^-5^ | 1.45 |
|  |  |  |  | Triglycerides | 3970 | -0.07 (0.02) | 0.0023 | 0.24 |
| 10 | rs730882082 | 19:11215991 (A) | Missense (*LDLR*) | LDL-cholesterol | 3809 | 0.66 (0.03) | 7.2x10^-99^ | 10.90 |
|  |  |  |  | Total cholesterol | 4342 | 0.55 (0.03) | 1.2x10^-78^ | 7.60 |
|  |  |  |  | ApoB | 1197 | 0.52 (0.06) | 2.0x10^-18^ | 6.18 |
|  |  |  |  | HDL-cholesterol | 4473 | -0.12 (0.03) | 7.8x10^-5^ | 0.35 |
|  |  |  |  | ApoA1 | 1197 | -0.12 (0.06) | 0.047 | 0.33 |
| 11 | rs429358 | 19:45411941 (C) | Missense (*APOE*) | LDL-cholesterol | 3809 | 0.20 (0.03) | 2.8x10^-12^ | 1.26 |
|  |  |  |  | Total cholesterol | 4342 | 0.17 (0.03) | 2.6x10^-11^ | 1.03 |
|  |  |  |  | HDL-cholesterol | 4473 | -0.13 (0.03) | 7.3x10^-7^ | 0.57 |
|  |  |  |  | ApoB | 1197 | 0.24 (0.05) | 1.3x10^-6^ | 1.92 |
|  |  |  |  | Triglycerides | 3970 | 0.11 (0.03) | 1.9x10^-4^ | 0.36 |

**Supplementary Table 4**. All lipid association signals (p<0.05) for the 11 loci with a genome-wide significant association signal. Loci number 1 and 2 are potentially novel and the remaining loci have been associated with lipid levels previously (3, 4). Effect sizes are reported as transformed (β_SD_) values and corresponding standard error (SE) estimates.

|  |  |  |  | | | **Greenlanders** | | | | | | **UK Biobank** | | | |
| --- | --- | --- | --- | --- | --- | --- | --- | --- | --- | --- | --- | --- | --- | --- | --- |
|  |  |  | **MAF (%)** | | | **LDL-cholesterol** | | | **Total cholesterol** | | | **LDL-cholesterol** | | **Total cholesterol** | |
| **#** | **rsID** | **posHg19** | **GR** | **EU** | **EA** | **β_SD_ (SE)** | **p-value** | **PVE (%)** | **β_SD_ (SE)** | **p-value** | **PVE (%)** | **β_SD_ (SE)** | **p-value** | **β_SD_ (SE)** | **p-value** |
| 1 | rs4927191 | 1:55491702T>C | 16.4 | 27.3 | 19.7 | -0.23 (0.03) | 1.2x 10^-12^ | 1.29 | -0.17 (0.03) | 2.3x10^-9^ | 0.83 | -0.03 (0.0024) | 6.8x10^-37^ | -0.027 (0.0024) | 1.4x10^-29^ |
| 2 | rs4609471 | 1:55493584C>A | 15.1 | 20.7 | 12.8 | -0.23 (0.03) | 2.7x 10^-12^ | 1.30 | -0.17 (0.03) | 8.9x10^-9^ | 0.76 | -0.032 (0.0026) | 5.6x10^-33^ | -0.028 (0.0027) | 2.4x10^-25^ |
| 3 | rs12117661 | 1:55487346C>G | 15.6 | 22.9 | 13.1 | -0.22 (0.03) | 6.5x 10^-12^ | 1.25 | -0.17 (0.03) | 1.1x10^-8^ | 0.74 | -0.029 (0.0024) | 9.1x10^-33^ | -0.025 (0.0024) | 1.5x10^-25^ |
| 4 | rs34232196 | 1:55489542C>T | 15.6 | 22.8 | 12.7 | -0.22 (0.03) | 6.8x 10^-12^ | 1.25 | -0.16 (0.03) | 1.5x10^-8^ | 0.72 | -0.029 (0.0024) | 1.5x10^-32^ | -0.025 (0.0024) | 1.9x10^-25^ |
| 5 | rs17111483 | 1:55485098T>C | 10.2 | 9.5 | 20.7 | -0.26 (0.04) | 7.8x 10^-12^ | 1.25 | -0.22 (0.03) | 2.8x10^-10^ | 0.91 | -0.03 (0.0035) | 1.1x10^-17^ | -0.028 (0.0035) | 7.5x10^-16^ |
| 6 | rs4500361 | 1:55490861T>C | 15.8 | 22.8 | 12.6 | -0.22 (0.03) | 5.1x 10^-11^ | 1.30 | -0.17 (0.03) | 5.2x10^-9^ | 0.84 | -0.028 (0.0024) | 1.3x10^-31^ | -0.025 (0.0024) | 8.5x10^-25^ |
| 7 | rs200159426 | 1:55491780A>G | 16.3 | 24.7 | 19.4 | -0.22 (0.03) | 5.6x 10^-11^ | 1.27 | -0.17 (0.03) | 2.0x10^-8^ | 0.82 | -0.03 (0.0024) | 2.4x10^-37^ | -0.027 (0.0024) | 5.0x10^-30^ |
| 8 | rs187607506 | 1:55491915C>T | 15.7 | 21.3 | 10.8 | -0.22 (0.03) | 1.3x 10^-10^ | 1.19 | -0.17 (0.03) | 2.0x10^-8^ | 0.79 | -0.028 (0.0025) | 9.3x10^-28^ | -0.024 (0.0025) | 1.1x10^-21^ |

**Supplementary Table 5.** Variants associated with LDL-cholesterol at p<1x10^-9^ from the independent *PCSK9* association signal. The minor allele frequencies in non-Finnish Europeans (EU) and East-Asians (EA) were obtained from the gnomAD data set v.2. The European association studies were based on 459,227 individuals from the UK Biobank and were conditioned on the *PCSK9* loss-of-function variant (rs11591147).

| **#** | **rsID** | **Tibial nerve** | | **Visceral adipose** | | **Lung** | | **Whole Blood** | | **Tibial artery** | | **Subcutaneous adipose** | |
| --- | --- | --- | --- | --- | --- | --- | --- | --- | --- | --- | --- | --- | --- |
|  |  | **NES** | **p-value** | **NES** | **p-value** | **NES** | **p-value** | **NES** | **p-value** | **NES** | **p-value** | **NES** | **p-value** |
| 1 | rs4927191 | -0.73 | 8.1x10^-52^ | -0.47 | 2.3x10^-14^ | -0.26 | 3.7x10^-16^ | -0.18 | 2.9x10^-11^ | -0.22 | 4.4x10^-6^ | -0.19 | 1.1x10^-4^ |
| 2 | rs4609471 | -0.79 | 1.3x10^-54^ | -0.45 | 8.8x10^-12^ | -0.28 | 3.0x10^-17^ | -0.19 | 6.6x10^-11^ | - | NS | - | NS |
| 3 | rs12117661 | -0.82 | 2.3x10^-61^ | -0.46 | 1.2x10^-12^ | -0.29 | 4.0x10^-19^ | -0.19 | 8.3x10^-11^ | -0.21 | 2.4x10^-5^ | -0.21 | 7.9x10^-5^ |
| 4 | rs34232196 | -0.83 | 3.7x10^-62^ | -0.46 | 1.4x10^-12^ | -0.30 | 5.4x10^-20^ | -0.19 | 7.3x10^-11^ | -0.22 | 1.8x10^-5^ | -0.20 | 9.5x10^-5^ |
| 5 | rs17111483 | -0.37 | 1.9x10^-6^ | -0.46 | 1.3x10^-7^ | - | NS | - | NS | - | NS | - | NS |
| 6 | rs4500361 | -0.83 | 2.5x10^-64^ | -0.44 | 1.6x10^-11^ | -0.30 | 4.4x10^-20^ | -0.18 | 4.0x10^-10^ | NS | NS | -0.22 | 2.7x10^-5^ |
| 7 | rs200159426 | NA | NA | NA | NA | NA | NA | NA | NA | NA | NA | NA | NA |
| 8 | rs187607506 | NA | NA | NA | NA | NA | NA | NA | NA | NA | NA | NA | NA |

**Supplementary Table 6.** Association between the variants from the association signal near *PCSK9* and PCSK9 expression across tissues with a p<2.0x10^-4^ for at least one of the variants in the GTEx portal (5). The variants are ordered according to their significance in the LDL-association signal. NA, not available; NES, normalized effect size; NS, not significant.

| **Association signals near *PCKS9*** | | **Greenlanders** | | | | | | | | **Europeans (UK biobank)** | | | | | | | |
| --- | --- | --- | --- | --- | --- | --- | --- | --- | --- | --- | --- | --- | --- | --- | --- | --- | --- |
|  |  | **Myocardial infarction** | | **Ischaemic stroke** | | **Revascularization procedures** | | **Any CVD** | | **Myocardial infarction** | | **Ischaemic stroke** | | **Revascularization procedures** | | **Any CVD** | |
| **#** | **rsID** | **HR (95% CI)** | **p-value** | **HR (95% CI)** | **p-value** | **HR (95% CI)** | **p-value** | **HR (95% CI)** | **p-value** | **HR (95% CI)** | **p-value** | **HR (95% CI)** | **p-value** | **HR (95% CI)** | **p-value** | **HR (95% CI)** | **p-value** |
| 1 | rs4927191 | 0.74 (0.52, 1.06) | 0.102 | 0.78 (0.61, 1.01) | 0.063 | 1.03 (0.71, 1.48) | 0.888 | 0.85 (0.70, 1.03) | 0.097 | 0.97 (0.95, 1.00) | 0.024 | 1.00 (0.96, 1.05) | 0.880 | 0.96 (0.93, 0.98) | 2.80x10^-4^ | 0.97 (0.96, 0.99) | 0.0038 |
| 2 | rs4609471 | 0.78 (0.54, 1.13) | 0.190 | 0.77 (0.59, 1.01) | 0.057 | 1.05 (0.72, 1.52) | 0.805 | 0.86 (0.70, 1.05) | 0.131 | 0.99 (0.96, 1.01) | 0.347 | 1.01 (0.96, 1.06) | 0.755 | 0.96 (0.93, 0.99) | 0.0024 | 0.98 (0.96, 1.00) | 0.035 |
| 3 | rs12117661 | 0.75 (0.52, 1.08) | 0.127 | 0.79 (0.61, 1.02) | 0.070 | 1.00 (0.69, 1.46) | 0.993 | 0.85 (0.70, 1.04) | 0.107 | 0.98 (0.95, 1.00) | 0.053 | 1.01 (0.97, 1.05) | 0.730 | 0.96 (0.93, 0.98) | 3.10x10^-4^ | 0.97 (0.96, 0.99) | 0.0047 |
| 4 | rs34232196 | 0.75 (0.52, 1.09) | 0.130 | 0.78 (0.60, 1.01) | 0.057 | 1.00 (0.69, 1.46) | 0.985 | 0.84 (0.69, 1.03) | 0.094 | 0.98 (0.95, 1.00) | 0.047 | 1.00 (0.96, 1.05) | 0.840 | 0.96 (0.93, 0.98) | 3.40x10^-4^ | 0.97 (0.95, 0.99) | 0.0044 |
| 5 | rs17111483 | 0.66 (0.41, 1.06) | 0.086 | 0.90 (0.67, 1.20) | 0.464 | 1.26 (0.83, 1.92) | 0.275 | 0.92 (0.73, 1.16) | 0.506 | 0.98 (0.95, 1.02) | 0.304 | 0.98 (0.92, 1.04) | 0.470 | 0.98 (0.94, 1.01) | 0.185 | 0.99 (0.96, 1.02) | 0.390 |
| 6 | rs4500361 | 0.80 (0.55, 1.17) | 0.249 | 0.79 (0.60, 1.03) | 0.082 | 0.91 (0.61, 1.36) | 0.649 | 0.86 (0.70, 1.06) | 0.152 | 0.98 (0.95, 1.00) | 0.055 | 1.01 (0.96, 1.05) | 0.750 | 0.96 (0.94, 0.98) | 6.10x10^-4^ | 0.97 (0.96, 0.99) | 0.0069 |
| 7 | rs200159426 | 0.80 (0.55, 1.16) | 0.234 | 0.81 (0.62, 1.05) | 0.116 | 0.99 (0.67, 1.46) | 0.966 | 0.88 (0.72, 1.08) | 0.213 | 0.97 (0.95, 1.00) | 0.022 | 1.00 (0.96, 1.04) | 0.975 | 0.96 (0.93, 0.98) | 2.00x10^-4^ | 0.97 (0.95, 0.99) | 0.0032 |
| 8 | rs187607506 | 0.81 (0.56, 1.18) | 0.276 | 0.79 (0.61, 1.04) | 0.094 | 1.00 (0.67, 1.48) | 0.992 | 0.88 (0.71, 1.08) | 0.216 | 0.98 (0.95, 1.00) | 0.062 | 0.99 (0.95, 1.04) | 0.731 | 0.96 (0.94, 0.98) | 0.0012 | 0.97 (0.96, 0.99) | 0.0089 |

**Supplementary Table 7.** Association between the variants from the association signal near *PCSK9* (p<10^-9^) and different CVD outcomes in Greenlanders and in Europeans from the UK Biobank, analyzed using a model adjusted for age, sex, and the first 10 principal components. The analyses in Europeans were conditioned on the *PCSK9* loss-of-function variant (rs11591147) (6). CVD, cardiovascular disease; HR, hazard ratio.

|  | **N** | β (SE) | **β_SD_ (SE)** | **p-value** |
| --- | --- | --- | --- | --- |
| **Lipid profile** |  |  |  |  |
| LDL-cholesterol (mmol/L) | 3809 | -0.23 (0.04) | -0.22 (0.03) | 6.5x10^-12^ |
| Total cholesterol (mmol/L) | 4342 | -0.19 (0.03) | -0.17 (0.03) | 1.1x10^-8^ |
| Triglycerides (mmol/L) | 3970 | -0.01 (0.02) | -0.02 (0.03) | 0.657 |
| HDL-cholesterol (mmol/L) | 4473 | 0.04 (0.02) | 0.08 (0.03) | 0.0064 |
| Apolipoprotein B (g/L) | 1197 | -0.04 (0.01) | -0.19 (0.05) | 4.1x10^-4^ |
| Apolipoprotein A1 (g/L) | 1197 | 0.007 (0.02) | 0.03 (0.05) | 0.573 |
| **Glucose homeostasis** |  |  |  |  |
| HbA1C (%) | 4447 | -0.01 (0.01) | -0.03 (0.02) | 0.315 |
| Fasting plasma glucose (mmol/L) | 3557 | -0.01 (0.03) | 0.02 (0.03) | 0.443 |
| 2h plasma glucose (mmol/L) | 3322 | 0.03 (0.08) | 0.03 (0.03) | 0.397 |
| Fasting serum insulin (pmol/L) | 3555 | 1.13 (1.41) | -0.004 (0.03) | 0.915 |
| 2h serum insulin (pmol/L) | 3323 | 11.47 (8.09) | 0.04 (0.04) | 0.263 |
| Fasting serum C-peptide (mmol/L)) | 3555 | -0.10 (10.49) | -0.02 (0.03) | 0.461 |
| 2h serum C-peptide (mmol/L) | 3323 | 3.32 (35.82) | 0.01 (0.03) | 0.736 |
| Systolic blood pressure (mm Hg) | 3984 | -0.29 (0.54) | -0.02 (0.03) | 0.506 |
| Diastolic blood pressure (mm Hg) | 3984 | -0.19 (0.37) | -0.02 (0.03) | 0.585 |
| **Anthropometrics** |  |  |  |  |
| BMI (kg/m^2^) | 4447 | -0.13 (0.15) | -0.02 (0.03) | 0.439 |
| Weight (kg) | 4453 | -0.51 (0.44) | -0.03 (0.03) | 0.292 |
| Waist (cm) | 4420 | -0.15 (0.40) | -0.01 (0.03) | 0.803 |
| Waist-hip ratio | 4417 | -0.003 (0.002) | -0.04 (0.03) | 0.178 |

**Supplementary Table 8.** Associations between the potential causal variant in the *PCSK9* association signal (rs12117661) and lipid levels, measures of glucose homeostasis, and anthropometrics in Greenlanders. Effect sizes and corresponding standard errors (SE) are shown as untransformed (β) and quantile transformed (β_SD_) values. p-values were calculated based on the quantile transformed trait values.

|  |  | **Myocardial infarction** | | **Ischaemic stroke** | | **Revascularization procedures** | | **Any CVD** | |
| --- | --- | --- | --- | --- | --- | --- | --- | --- | --- |
| **Locus** | **variant ID** | **HR (95% CI)** | **p-value** | **HR (95% CI)** | **p-value** | **HR (95% CI)** | **p-value** | **HR (95% CI)** | **p-value** |
| 1 | rs78168195 | 1.07 (0.63, 1.80) | 0.806 | 1.07 (0.74, 1.53) | 0.731 | 1.42 (0.84, 2.38) | 0.188 | 1.15 (0.87, 1.53) | 0.325 |
| 2 | rs148015058 | 1.15 (0.66, 1.98) | 0.622 | 1.20 (0.77, 1.87) | 0.425 | 1.08 (0.56, 2.07) | 0.815 | 1.16 (0.83, 1.63) | 0.386 |
| 3 | rs4927191 | 0.74 (0.52, 1.06) | 0.102 | 0.78 (0.61, 1.01) | 0.063 | 1.03 (0.71, 1.48) | 0.888 | 0.85 (0.70, 1.03) | 0.097 |
| 4 | rs312029 | 0.79 (0.53, 1.18) | 0.253 | 0.83 (0.63, 1.09) | 0.186 | 0.58 (0.35, 0.98) | 0.042 | 0.81 (0.65, 1.02) | 0.072 |
| 5 | rs2169387 | 0.83 (0.58, 1.18) | 0.293 | 1.22 (0.99, 1.50) | 0.063 | 0.87 (0.59, 1.29) | 0.481 | 1.10 (0.92, 1.30) | 0.301 |
| 6 | rs287 | 0.89 (0.67, 1.17) | 0.399 | 0.97 (0.80, 1.18) | 0.747 | 1.00 (0.73, 1.35) | 0.980 | 0.94 (0.81, 1.10) | 0.445 |
| 7 | rs2519093 | 1.16 (0.87, 1.55) | 0.308 | 0.91 (0.74, 1.11) | 0.351 | 1.15 (0.83, 1.60) | 0.395 | 1.00 (0.85, 1.17) | 0.996 |
| 8 | rs187850276 | 0.96 (0.71, 1.30) | 0.798 | 1.04 (0.85, 1.26) | 0.733 | 0.91 (0.65, 1.29) | 0.609 | 1.04 (0.89, 1.23) | 0.600 |
| 9 | rs8045855 | 1.09 (0.83, 1.42) | 0.535 | 1.07 (0.90, 1.28) | 0.444 | 1.02 (0.76, 1.38) | 0.882 | 1.05 (0.91, 1.21) | 0.507 |
| 10 | rs730882082 | 1.39 (0.99, 1.95) | 0.060 | 1.12 (0.89, 1.42) | 0.334 | 1.73 (1.21, 2.48) | 0.0026 | 1.25 (1.03, 1.50) | 0.022 |
| 11 | rs429358 | 0.93 (0.68, 1.27) | 0.646 | 0.92 (0.74, 1.14) | 0.427 | 1.22 (0.89, 1.68) | 0.217 | 0.97 (0.82, 1.15) | 0.740 |

**Supplementary Table 9.** Association analysis of lead variants of the 11 association signals and different cardiovascular outcomes in Greenlanders using a Cox-regression model adjusted for age, sex, and the first 10 principal components. CVD, cardiovascular disease; HR, hazard ratio; IS, ischemic stroke; MI, myocardial infarction; RP, revascularization procedures.

|  |  |  | **PVE (%)** | | | | | |
| --- | --- | --- | --- | --- | --- | --- | --- | --- |
|  |  |  | **GRS** | | **Age** | | **BMI** | |
| **GRS/Trait** | **Beta (SE)** | **p-value** | **model 1** | **model 2** | **model 1** | **model 2** | **model 1** | **model 2** |
| Triglycerides | 0.64 (0.07) | 4.5x10^-22^ | 2.35 | 1.92 | 0.02 | 0.12 | 12.3 | 12.0 |
| Total cholesterol | 0.91 (0.04) | 3.0x10^-123^ | 12.2 | 9.98 | 11.9 | 10.9 | 2.25 | 3.01 |
| LDL-cholesterol | 0.88 (0.03) | 6.2x10^-148^ | 16.3 | 13.7 | 6.44 | 5.91 | 5.93 | 5.86 |
| HDL-cholesterol | 0.43 (0.05) | 1.8x10^-15^ | 1.42 | 1.31 | 6.27 | 3.41 | 14.2 | 11.6 |
| Apolipoprotein A1 | 0.27 (0.11) | 0.012 | 0.53 | 0.73 | 1.82 | 0.87 | 5.48 | 4.69 |
| Apolipoprotein B | 0.91 (0.07) | 1.4x10^-32^ | 11.3 | 10.8 | 0.26 | 0.74 | 6.05 | 6.29 |

**Supplementary Table 10.** Effect size and p-value for association analyses in Greenlanders adjusted for age, sex, BMI, and the first 10 principal components, and partial variance explained (PVE) for each genetic risk score (GRS), age, and BMI adjusted for age, sex, BMI, and the first 10 principal components (model 1), or the first 10 principal components (model 2).

References

1. Graham SE, Clarke SL, Wu KH, Kanoni S, Zajac GJM, Ramdas S, et al. The power of genetic diversity in genome-wide association studies of lipids. Nature. 2021;600(7890):675-9.

2. Trinder M, Francis GA, Brunham LR. Association of Monogenic vs Polygenic Hypercholesterolemia With Risk of Atherosclerotic Cardiovascular Disease. JAMA Cardiol. 2020;5(4):390-9.

3. Dube JB, Wang J, Cao HN, McIntyre AD, Johansen CT, Hopkins SE, et al. Common Low-Density Lipoprotein Receptor p.G116S Variant Has a Large Effect on Plasma Low-Density Lipoprotein Cholesterol in Circumpolar Inuit Populations. Circ-Cardiovasc Genet. 2015;8(1):100-U90.

4. Surakka I, Horikoshi M, Magi R, Sarin AP, Mahajan A, Lagou V, et al. The impact of low-frequency and rare variants on lipid levels. Nature Genet. 2015;47(6):589-97.

5. GTEx Consortium. The Genotype-Tissue Expression (GTEx) pilot analysis: Multitissue gene regulation in humans. Science. 2015;348(6235):648-60.

6. Bycroft C, Freeman C, Petkova D, Band G, Elliott LT, Sharp K, et al. The UK Biobank resource with deep phenotyping and genomic data. Nature. 2018;562(7726):203-9.
